# Aggregate excess demand on wall street

**DOI:** 10.1016/j.heliyon.2021.e08355

**Published:** 2021-11-11

**Authors:** Qingyuan Han, Steve Keen

**Affiliations:** a University of Alabama in Huntsville, Department of Atmospheric Science, USA; b Kinston University in London, School of Economics, History and Politics, UK

**Keywords:** Aggregate excess demand, Prediction of market directions, Market maker inventory, Walrasian general equilibrium, Utility maximization, Perfect competition, Asset market price change, Efficient market hypothesis, Behavioral finance theory, Financial market crises

## Abstract

The rational investor behavior and news triggered price change assumed by the Efficient Market Hypothesis (EMH) could not explain most of asset price variances_,_ suggesting the need for an alternative theory. The Behavioral Finance Theory (BFT) advocates those economic judgments and decisions in markets are often irrational because of systematic and predictable psychological bias. However, due to the lack of measurable investment behaviors, proponents of the efficient market hypothesis argue that irrational behavior could not be reliably identified and predicted. Here we show that the price-takers behavior gauged by the normalized excess demand (NED) can be measured and the results explain most of the variances of SP500 daily returns over eight years of available data, the remaining variances are due to price-makers behavior, an influence abstracted out by the Walrasian general equilibrium theory. The interactions between behaviors of price-takers and price-makers drive market price fluctuations. For short-term prediction, we demonstrate that detected market makers' inventory positions often lead to intraday and daily market reversals. For long-term forecasting, feedback analyses of NED and SP500 data reveal signals of looming plunges and recovery processes in 2000, 2008, and 2020 market crises.

## Teaser

Financial market; Efficient market; Behavioral economics; Financial crises; Aggregate excess demand; Prediction of market directions; Market maker inventory.

## 1 Introduction

Two core premises set the basis of Neoclassical economics (Thaler, 2015): constrained utility maximization (Jevons, 1871) for microeconomics and the Walrasian general equilibrium theory (Walras, 1900) for macroeconomics. The utility is an ordinal measure of satisfaction, pleasure, or happiness (Bentham, 1781). The unsettled state of ideas on utility maximization is manifest in two opposing perspectives among economists, both of which have been awarded the Nobel Prize in economics^1^. At one end, proponents of the efficient market hypothesis (Fama, 1970) believe that utility maximization is realized through rational behaviors of investors based on expectations about intrinsic values (von Neumann and Morgenstern, 1944; Fama and French, 2004). Although EMH dominated academic finance around the 1970s, the findings that it could not explain most of the volatility (Shiller 2003) and the asset price variances of the stock markets (French and Roll, 1986; Roll, 1988; Cutler et al., 1989) suggest the need for an alternative theory. At the other end, advocators of behavior finance theory assert that predictable irrational decisions (Ariely, 2008) influenced by market psychology play a major role in market instability causing bubbles and crises (Tversky and Kahneman, 1974; Akerlof and Shiller, 2009). In defending the EMH, Fama argued that irrational behaviors are neither identified nor reliably predicted from historical market data (Fama, 1998, 2014; Chicago Booth Review, 2016). Indeed, without information on investor behaviors, it is implausible to tag price movements as consequences of rational or irrational behaviors, let alone predict future movements. Procurement of such information is theoretically formidable because the aggregate excess demand function, a gauge of market behaviors, may take arbitrary forms from the assumption of maximizing individual utilities, as proven by the Sonnenschein–Mantel–Debreu (SMD) theorem (Sonnenschein, 1973a; Mantel, 1974; Debreu, 1974).

The Walrasian general equilibrium theory assumes that market equilibrium is established through “groping”, and would be constantly reestablished after being disturbed (Walras, 1900, p319). However, the theory's repeated failures to explain and predict market crises (Taleb, 2007; Derman and Wilmott, 2009), raised serious questions about its validity (Ackerman, 2002; Lavoie, 2015). In contrast, Keynes (1936) contended that the market boom and the following collapse were due to uncertain foresight about the future. Akerlof (1970) showed that asymmetric information may lead to market collapse. Minsky (1975) argued that the capitalist economy is “*not a self-correcting system*” because it “*contains the potential for runaway expansion*.” It has recently been shown that Minsky's Financial Instability Hypothesis (Minsky, 1992) can be derived from macroeconomic definitions, making it arguably better grounded in macroeconomics than general equilibrium theory is in microeconomics (Keen, 2020). The Walrasian equilibrium model cannot generate systemic instability because its abstractions pruned essential features of real markets (Minsky, 1982; Keen, 2017) that left no room for either credit and feedback processes, two crucial mechanisms in historical deep depression and inflationary episodes (Friedman and Schwartz, 1963; Vague, 2019). Economists have studied the behavior interactions and feedbacks from descriptive perspectives such as the herding effect (Thaler and Sustein, 2008; Nofsinger and Sias, 1999), reflexivity (Soros, 1994), adaptive markets (Lo, 2017), and narrative economics (Shiller, 2019). Nonetheless, quantitative understanding of the feedback process has not yet been attained, because the behaviors of market participants has not been measurable thus far.

Identifying data that describes the aggregate behaviors of investors and analyzing behavior interactions and feedbacks in a manner that enables these competing theories to be assessed is necessary to advance our understanding of financial economics. This essay reports our efforts in these regards. We have developed a technique to measure aggregate excess demand in a manner that maximizes power to explain daily SP500 returns, the most commonly used index gauging the performance of the U.S. stock market (Zhang et al., 2016). The real-time measured normalized excess demand (NED) can explain most of the variance of the SP500 daily returns over eight years (2153 observations and counting), which is consistent with recent findings in the literature (Johnson and Watson, 2018) but in a more quantitative way. Six signals of behavioral feedbacks between demand and supply of market liquidity that are indicative of future movements are summarized from historical data. For short-term prediction, we found market makers’ inventory problems are associated with intraday and daily market fluctuations. For the long-term forecast, the six signals can serve as guidance indicating consents or conflicts of market participants that shed light on market future directions such as in the plunges and recovery processes in 2000, 2008, and 2020 market crises.

## 2 Model

Paul Samuelson (1941, Eq. 11) mathematically formulated Walras's law of supply and demand that the price changes at a rate proportional to the excess demand by, $\frac{dp}{dt}=H\left[D\left(p\right)-S\left(p\right)\right]$, where p is the price, D(p)-S(p) is the excess demand, and H (0) = 0, H’>0. This, however, is a model of a market mechanism of price takers in which price-making has no role (Arrow, 1959; Bradfield and Zabel, 1979; Bryant, 1980): it is only “the impersonal forces of the market.” (Scitovsky, 1952) In stock markets however, transactions are executed at stock quotes, the bid price and the ask price, which are determined by limit orders. Limit order users are acting as price-makers or liquidity providers whose behaviors have significant impacts on stock price adjustments, which is not accounted for by Walrasian excess demand alone (Asparouhova et al., 2003). Detailed market data analyses show that limit order revisions caused stock price change more often than trades (Easley et al., 2012). Consequently, an imbalance between buying and selling sides in limit order books is indicative of the directions of future market movements (Lipton et al., 2014; Gould and Bonart, 2016). A significant disparity of limit-sell and -buy orders led to the market flash crash on May 6, 2010 (CFTC and SEC, 2010). Hence, the omission of the roles of price-makers in Walrasian theory precludes interactions and feedbacks between price-takers and price-makers to generate instability. We add the term M to the price adjustment equation to mend this problem.

The Walrasian demand has to be homogeneous of degree zero (Mas-Colell et al., 1995). Without loss of generality, we rewrite the equation as:

(1) $$\frac{dlnp}{dt}=H\left[\frac{D-S}{D+S}\right]+M$$

where (D-S)/(D + S) is NED that describes the aggregate behavior of liquidity-takers, and M the behavior of liquidity-suppliers. We adopt an approach to retrieve NED based on Eq. (1) by maximizing the explanatory power of NED to daily SP500 returns. The approach is conducted through a trial-and-error process, as described in the methodology section, to obtain NED for six time-horizons: 5-minute, 15-minute, hourly, daily, weekly, and monthly. Based on the available data we have, the three intraday NEDs are from April 30, 2013, to the present and the other three NEDs cover the period of 1999-present.

**Figure 1:**
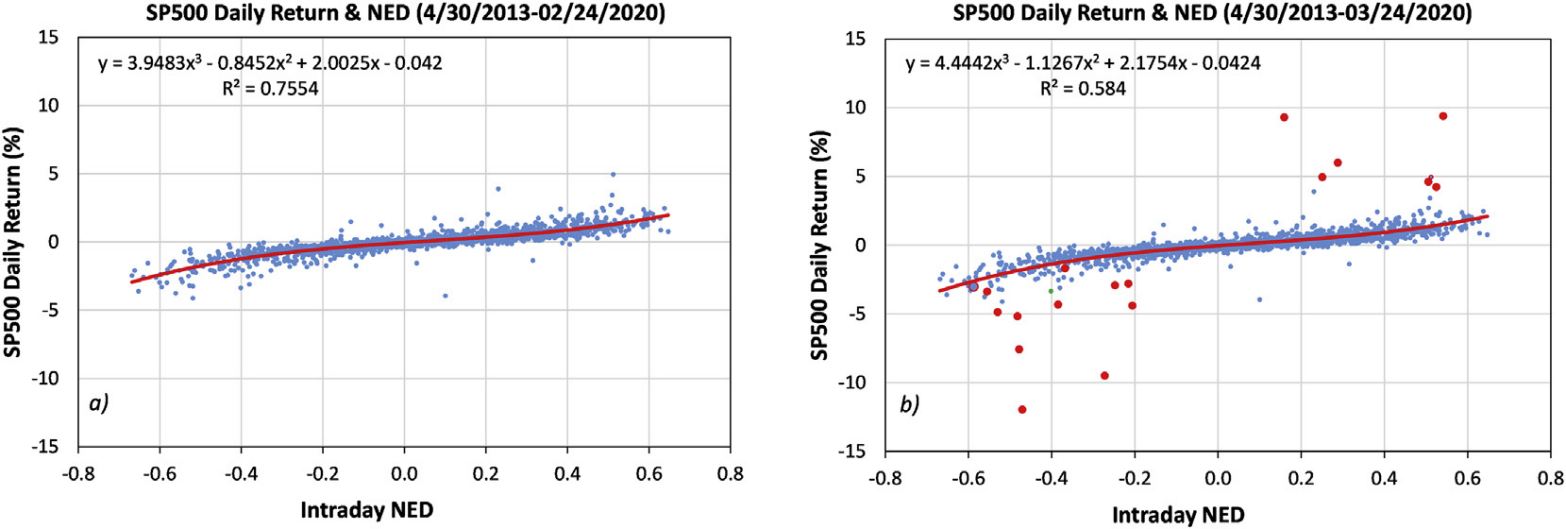
Scatter plots of intraday NED (daily average of 5-minute NED) and SP500 daily returns for a) 4/30/2013-2/24/2020 and b) 4/30/2013-3/24/2020. The extended one-month period in b) includes 4 circuit breaker triggered events. 18 of the extended-period data are marked by red dots.

Since large price changes are associated with illiquidity caused by order revision and cancellations (Farmer et al., 2004; Weber and Rosenow, 2006), which are behaviors of price-makers, we expect that the explanatory power of market fluctuations by NED would be reduced during a highly volatile period. This is exactly what we have found in the relation between SP500 daily return and the third-order polynomial of intraday NED shown in Figure 1. For 1716 trading days from April 30, 2013, to February 24, 2020, R^2^ is 75.54%, suggesting that three-quarters of variances of the SP500 daily returns could be explained by the behavior of price-takers in relatively nonvolatile markets.

However, extending the period by merely 21 days to March 24, 2020, reduced the R^2^ value dramatically to 58.40%. This extended period includes the impact of the panic surrounding the COVID-19 pandemic when market circuit breakers were triggered four times, which was unprecedented, even in comparison to the two market crises in 2000 and 2008.

The intraday NED shown in Figure 1 is the daily average of all 5-minute NEDs (see next section for calculation method). The explanatory power of NED decreases when the time horizon is broadened. For daily averages of 15-minute and hourly NEDs, the R^2^ over the same period as in Figure 1a) reduce to 68.32% and 64.78%, respectively. For the daily NED, the R^2^ is further curtailed to only 13.59%. These results are consistent with the findings by Blume et al. (1989) and Chordia et al. (2002). The former found that the correlation between 15-minute return and market order imbalance is 0.81–0.86 (R^2^ 0.66 to 0.74), and the latter reported that the R^2^ by regression between SP500 daily return and three variables (including market order imbalance) is 0.247.

While a mathematical restriction helps generate a variable with maximum explanatory power, the caveat of such an approach is that the physical meaning of the resultant variable might be ambiguous, similar to that of the Empirical Orthogonal Function (EOF) or the Principal Component Analysis (PCA). In our case, both NED and M have impacts on market returns (Eq. 1), the explanatory power might be attributed to the combination of NED and M. The possible ambiguity behooves us to validate that our NED measures the aggregate behavior of price-takers. The logic is that when NED = 1 (−1), all price-takers are buying (selling), so that market makers are obligated to sell (buy) to supply liquidity and unwillingly accumulate short (long) inventory. Hence, the inventory positions of market makers can be revealed by the price range when NED = ±1. Due to their limited funds, market makers have to unwind these inventories at a price better than that when the inventories were accumulated so that they can continue to supply immediate liquidity (Garman, 1976; Amihud and Mendelson, 1980; Hendershott and Seasholes, 2007).

Therefore, if our retrieved NED describes the aggregate behaviors of price-takers, we can reveal the inventory positions of market makers when NED = ±1 and forecast future market reversals. We tested the idea using historical intraday data from May 2013 to April 2020 and found that over 90% of the time within 7 trading days of the inventory fill, the market returned to the inventory price or better, consistent with the model findings reported in the literature (Madhavan and Smidt, 1993). The test results are listed in Table 1.

**Table 1:** The success rate of unwinding market makers’ inventories within 7 days during 2013–2020.

Year	NED = +100%			NED = -100%		
	Short Inventory	Unwound	%	Long inventory	Unwound	%
2013	202	180	89	181	170	94
2014	223	199	89	199	189	95
2015	207	189	91	215	197	92
2016	197	176	89	157	146	93
2017	91	84	92	67	62	93
2018	216	195	90	201	179	89
2019	177	159	90	130	127	98
Jan–Apr,2020	98	92	90	105	98	93
**Total**	**1411**	**1274**	**90**	**1225**	**1168**	**93**

Based on this finding we launched an experiment of real-time market reversal forecasting started on May 8, 2020, with specified target prices in a finance forum^2^. We use SPY price because its return is roughly in line with that of the SP500. For significance, we only posted reversal price targets when SPY price was moving away from the target for more than $1 (about 10 points of SP500 index) although it would trim the success rate since all fulfilled small reversals were removed in the statistics. Our first forecast on May 8 was a 3.56% drop within three days when the market just finished a week with a 3.5% surge. The forum participants deemed the forecast highly implausible because the market kept on rising the next trading day. On the third day, however, they gaped in awe when the SPY price plunged to the predicted target without any news release and started to take the forecast seriously since. In the two-month experiment, we predicted 54 reversal targets in 44 trading days with a 90.75% success rate (49 targets reached within the specified time), including several large market surges and plunges in a single day not associated with any breaking political or economic news. The success rate of real-time market reversal forecasting suggests that NED represents the aggregate behavior of price-takers and that rewinding of market makers’ inventory is a contributing factor regulating intraday and day-to-day market reversals (Panayides, 2007).

## 3 Methodology

Investors behave differently in stock markets by placing orders: price-takers use market-orders (or executable limit orders), and price-makers submit limit orders. Many studies using historical data focused either on the behaviors of price-takers (Chakrabarty and Zhang, 2012) or on the actions of price-makers (Cont et al., 2014). These investigations are successful in revealing the market microstructure as the result of investor behaviors.

The SMD theorem proved that market demand cannot be inferred from the utility hypothesis (Sonnenschein, 1973b; Shafer and Sonnenschein, 1982) that has had long and profound impacts on economics (Rizvi, 2006). In the proof, however, the market prices are based on the implicit assumption of perfect competition (Walras, 1900; Robinson, 1934), despite the assumption's well-known weaknesses (Keynes, 1890; Knight, 1921; Chamberlin, 1962). These are even more evident in stock markets, where transaction prices are determined by the bid-ask spread of limit-orders placed by market makers who cannot be modelled as competitive actors, and which are frequently revised and canceled due to updated information to prevent loss and maximize profit (Corwin and Lipson, 2000; Calcagno and Lovo, 2006). In practice, aggregate behaviors of price-takers, as a measure of market demand, had been numerically observed for different horizons through calculations of market-order imbalance (Blume et al., 1989; Chordia et al., 2002). Our goal is to fit the numerical values of market-order imbalance into a function of price change that maximizes its explanatory power of SP500 daily return changes and can be measured in real-time. The approach is divided into three-steps. First, measure NED using a classification rule described below so that NED describes the aggregate behaviors of price-takers. Second, fit the numerical values of NED into an empirical function of the price through a trial-and-error process to maximize the explanatory power to SP500 daily return changes. The third step is to verify that the NED represents the behavior of price takers since the fitted empirical function may include contributions from price-makers due to the possible errors introduced in the first two steps. The results of the third step have been described in the Model section.

The expression of NED(p) is [D(p)-S(p)]/[D(p)+S(p)], where D(p) is the buying volume and S(p) the selling volume at a given price p that can be obtained from the market-order information. However, such information is not disclosed to the public based on NYSE OpenBook specifications (Baruch, 2005). Scholars have developed several algorithms for classifying trades as the buyer or seller-initiated transactions to study investor behaviors (Lee and Ready, 1991; Lee and Radhakrishna, 2000) and market microstructures (Chakrabarty and Zhang, 2012), each with different pros and cons due to complex market conditions and data recording procedures (Easley et al., 2012). We chose the tick rule as recommended by Finucane (2000) using the data downloaded from Scottrade. The absolute accuracy of the classification rule is not critical because the results are used as the data pool for finding an empirical function of normalized excess demand that will maximize the explanatory power of fluctuations in SP500 daily returns.

For a single stock, the empirical function Z is the inverse function of H in Eq. (1), so that an input of return change for each time step to the empiric function will generate a NED value for the time horizon. The dataset used for searching the empirical function includes five months of tick-by-tick data from 381 SP500 stocks selected following the rules of Chordia et al. (2002). The maximized adjusted R-squared for the 107 trading days in early 2013 is 0.8135. The high value of R-squared during the smooth market of these months reduced to 0.7546 before February 2020 after several market adjustment of 10% or more in October 2014, August 2015, February 2018, and December 2018. For the aggregate market, NED is the weighted average of NEDs of all component stocks. Then we adjust the parameters of the empirical function until they maximized the explanatory power for fluctuations of SP500 daily returns. We measure NED values in real-time, updated four times per minute.

## 4 Signals of behavior interactions and feedbacks

Theoretical studies of behavior interactions and feedbacks include information impact (Kyle, 1985; Holden and Subrahmanyam, 1992; Madhavan, 1992), and herding effect (Shiller, 1984; De Long et al., 1990; Nofsinger and Sias, 1999). However, it is difficult to identify these interactions from price data, especially in real-time. In stock markets, price-takers place market orders (or executable limit orders) that demand liquidity and price-makers submit limit orders that provide liquidity (Hopman, 2007). Since market price changes are caused by interactions of price-takers and price-makers, we can analyze the behavioral interactions once the aggregate behaviors of price-takers are measured by NEDs. We have summarized six signals that can identify behavioral interactions and infer future market directions.

The six signals are:

1. Uptrend signal: the ridges and troughs of NED are higher than the previous adjacent ones during market oscillations, and so too the peaks and valleys of SP500. The signal indicates that both price-takers and price-makers are optimistic about the market.
2. Downtrend signal: the ridges and troughs of NED are lower than the previous adjacent ones during market oscillations, and so too the peaks and valleys of SP500. This signal appears when most market participants are pessimistic.
3. Impending uptrend signal: the trough of NED is lower than the prior adjacent trough when the corresponding valley of SP500 is higher than the previous one. This is because the increased selling pressure cannot suppress SP500 due to a large volume of limit-buy orders at a higher level than the previous low. The signal reveals the market microstructure remarked in Arrow (1959), which has been used to predict market directions (Gould and Bonart, 2016; Cartea et al., 2018).
4. Looming downtrend signal: the ridge of NED is higher than the preceding adjacent ridge, while the corresponding peak of SP500 is lower than the previous one. This is a signal similar to signal 3 but in an opposite direction.
5. A downturn signal at an all-time market high: NED value is significantly below 100% at an all-time market high. The low NED value reflects that massive selling activities occur at market peaks, revealing actions of informed traders before the spread of private information.
6. A recovering signal at a new market low: NED value is above -100% when SP500 reaches a new low level. The raised NED value indicates that informed investors start to buy at a low price.

The first and second signals reflect positive feedbacks between price-takers and price-makers that may lead to overreactions and market instabilities. Signals 3 and 4 reveal different perceptions and behaviors of market participants that cause an imbalance in limit-order books. The last two signals are related to market sentiment reversals after excessive optimism and pessimism (De Bondt and Thaler, 1985; Spyrou, 2012).

## 5 Short-term prediction

It is a consensus that while there is some predictability for medium and long-term market reversal, there is no predictability at all in the short-term (intraday and daily) horizon (Subrahmanyam, 2005). We have shown short-term predictability based on the market makers' inventory problem. In this section, we will show that short-term market directions are inferable by the six signals.

### 5.1 Intraday fluctuations

**Figure 2:**
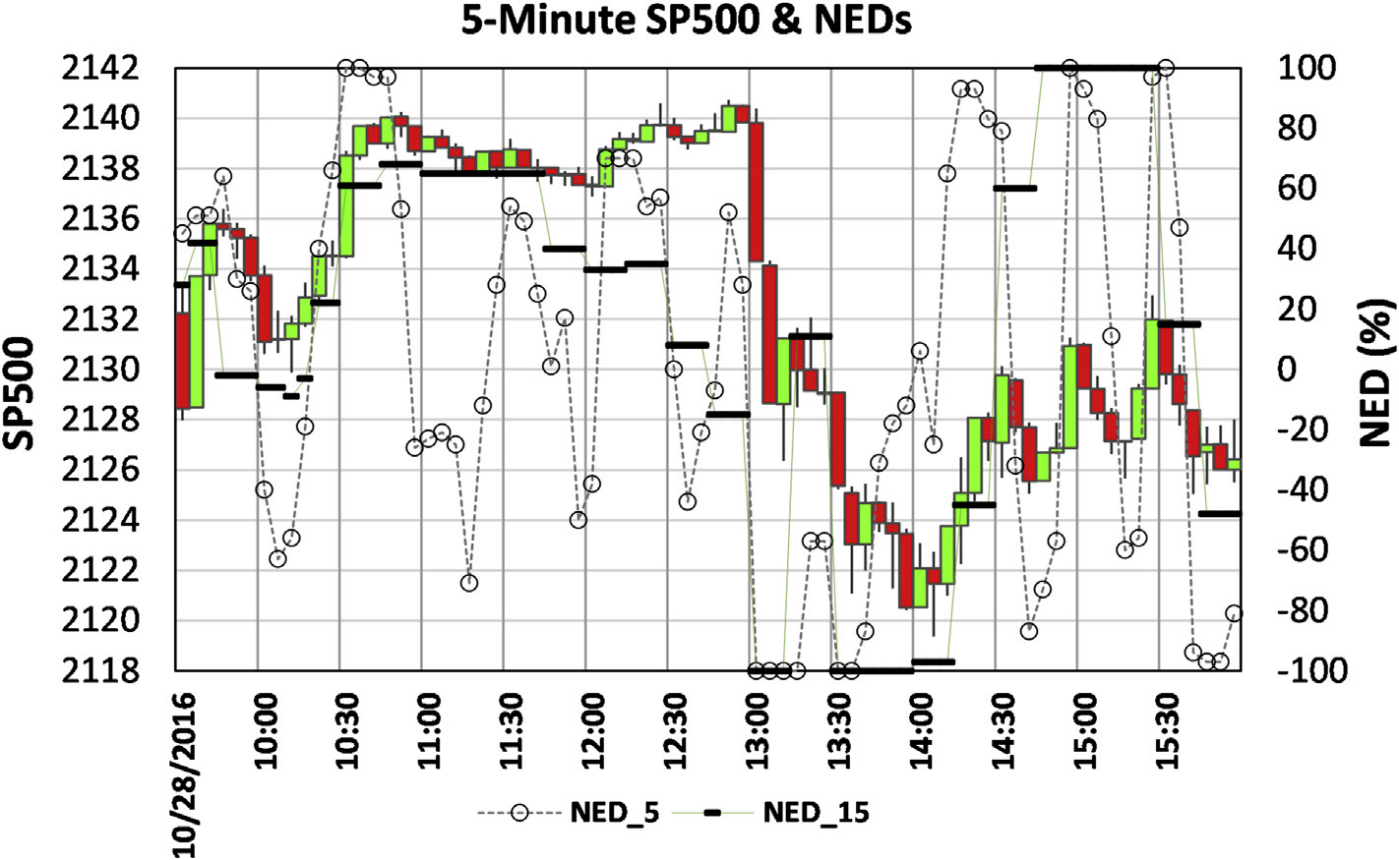
SP500 Intraday fluctuations on Oct. 28, 2016, when breaking news hit the market. Market movements can be explained and foreseen by applying the six rules using 5- and 15-minute NEDs.

Figure 2 shows SP500 on Oct. 28, 2016, at five-minute intervals with NED_5 (hollow circle) and NED_15 (short bar) for five- and fifteen-minute horizons, respectively.

Before 10:30, the market was in an uptrend from signal 1. NED-5 reached +1, and NED-15 was highest around 10:30 when SP500 rose to the peak, suggesting that price-takers and price-makers were both euphoric. Between 10:30 and 12:30, SP500 moved sideways, and NED_5 was oscillating, but NED_15 started decreasing. When SP500 reached the all-time high of the day around 12:50, NED_5 and NED-15 were not at their high points as they did around 10:30, implying conflict behaviors among traders. The decreasing NED_5, especially NED_15, reveals that increased sell orders were submitted (signal 5) as informed traders placing market orders for immediate execution (Linnainmaa, 2010). Then, the market plunged and turned into a downtrend identified by signal 2. When SP500 reached the lowest level at 14:05, the NED_5 was -0.25, way above -1, flagging the downtrend termination based on signal 6. The following three ridges of NED_5 identify an uptrend from signal 1 and the two troughs of NED_5 foresee an uptrend based on signal 3.

**Figure 3:**
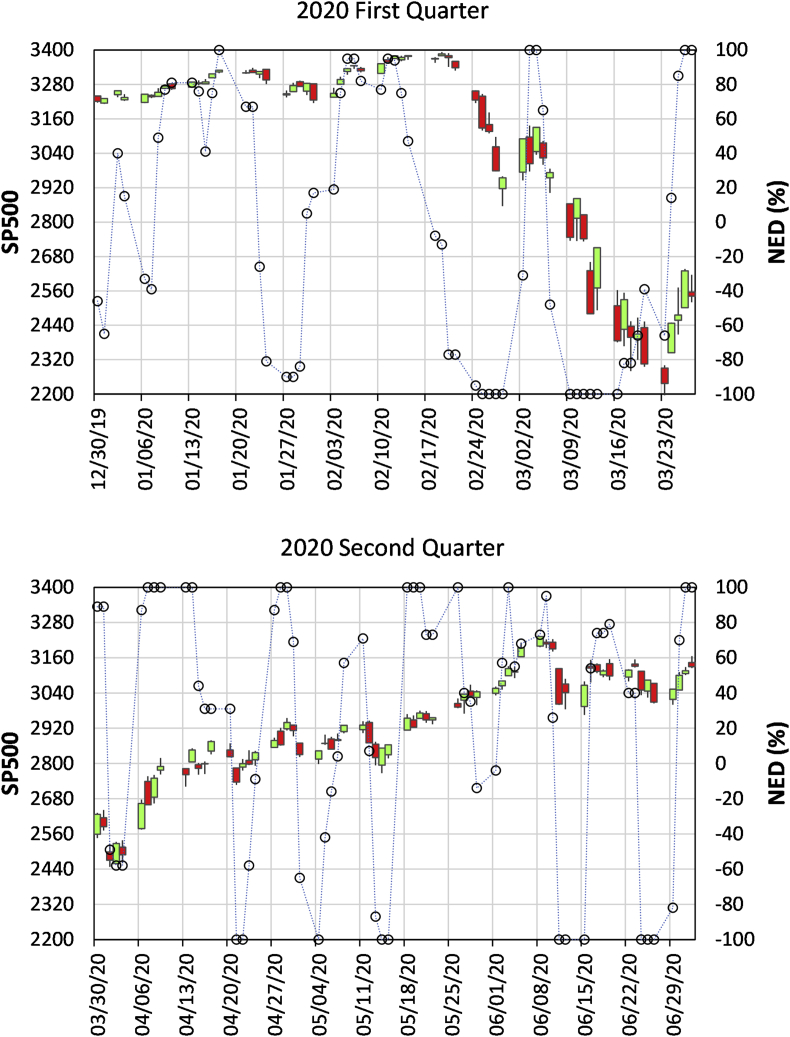
Daily SP500 and NED of the first (up panel) and second (lower panel) quarter of 2020. Labeled dates are Mondays of each week.

The market drop that started at 13:00 was due to breaking news about reopening the investigation case on Hillary Clinton's email by the FBI. Before being published by TV and Market Watch after 13:14, the information was released at 11:57 on Twitter by Jason Chaffetz^3^, who was the chair of the Committee on Oversight and Government Reform at that time. NEDs reflect behaviors of traders who have short-horizon information and submit market orders for an immediate execution (Harris, 1998; Kaniel and Liu, 2006). The continuous decrease of NED_15 means that the spread of information caused massive selling before the general public was notified through more popular news channels. The same information spreading process is recorded in a longer time-horizon before the market collapse due to the COVID-19 virus spread, which is shown in Figure 3.

Figure 2 provides an example illustrating the loading and unwinding process of market makers’ inventory positions. At 10:00 am, NED_5 reached +1, meaning that all price-takers were buying. The market makers had to sell and stack short-inventory at the price range, which was cleared at 13:00 when the market went down. Similarly, market makers had to pile up a long-inventory to buy when the NED was -1 at 13:05–13:15 and 13:30–13:35. The long-position of 13:30–13:35 was cleared up at 14:25, while the long-inventory filled at 13:05 was unwound the next day. At 14:55 and 15:35, two more short-inventory positions were loaded and cleared within half an hour.

### 5.2 Daily changes

Figure 3 shows the market fluctuation of 2020 under the influence of pandemic COVID-19.

Before January 17, the market was in an uptrend by signal 1. The trend reversed to a downtrend by comparing NED troughs and SP500 valleys between 1/27 and 1/15 based on signal 2. The uptrend was resumed by analysis between 2/5 and 2/11 from signal 3.

The market reached an all-time high on 2/19 when a signal-five appeared, warning of a market trend reversal because the daily NED was -0.13. The gradual decreasing NED started on 2/13, when Richard Burr, then chairman of the Senate Intelligence Committee, sold a large volume of stocks after receiving classified briefings on the COVID-19 outbreak. He informed his brother-in-law to do the same^4^. Then, the private information spread out so that selling actions snowballed, revealed by decreasing NED values for several days. A sudden market slide occurred on 2/24, and we can identify a trend reversal based on signal-two. On 3/3, after an SP500 bounce-back of more than 150 points in two days, the NED arrived at +1. However, the SP500 peak was significantly lower than that at the previous NED ridge, which was a signal-four warning of a looming downtrend.

An SP500 plunge of 900 points followed until 3/23. At the new SP500 valley on 3/23, the NED was -0.66, a signal-six indicating a possible market reversal. The trend reversal was confirmed on 3/26 by signal-one. The uptrend continued until 6/9 by signals one and three alternatively. On 6/11, the downtrend resumed by signal-two.

One important factor contributing to short-term reversals is the market makers’ inventory problem. For example, at the market low on 3/23, there was only one inventory long position of market makers at SP500 2364, much higher than the market close of 2237.40. The stimulus measures announced by the Federal Reserve led to a surge of 9.38% on 3/24, which helped to rewind the long-position. Similarly, on 6/10, when the market closed at SP500 3190.14, market makers had only short-positions at SP500 3065 or lower, as forecasted in our real-time prediction experiment^5^. The market plunged by 5.9% the next day without any associated breaking news.

## 6 Long-term prediction

One challenge that remains in financial economics is how to reconcile the unpredictability in the short-term, which has been long perceived as a random-walk process (Malkiel, 2019), and predictability for the long run based on macro variables such as dividend yield and short interest (Ang and Bekaert, 2007). In this section, we show that the six signals working in the short-term fluctuations can also be applied to long-term variations to identify trends and anticipate looming market reversals.

### 6.1 The dot-com bubble of 2000

**Figure 4:**
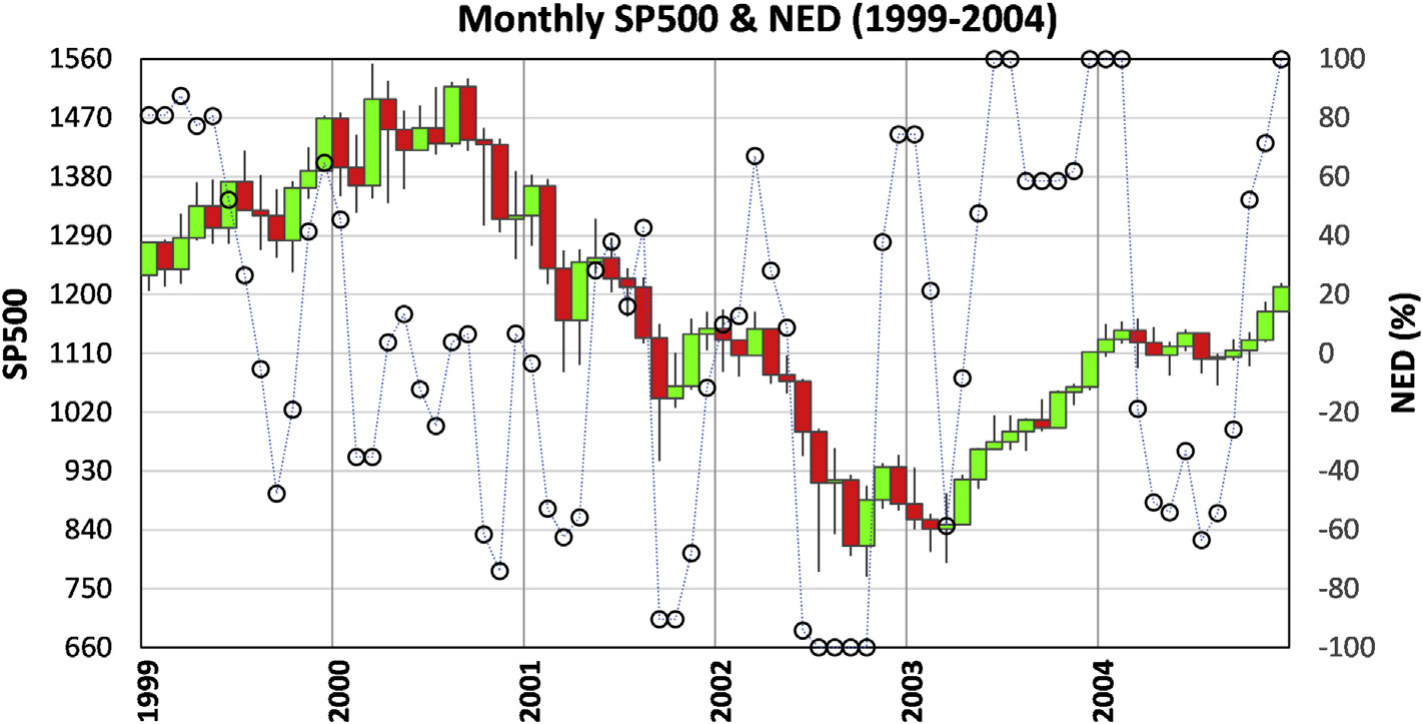
Monthly SP500 and NED for 1999–2004 that covers the period of the onset and recovery of the 2000 dot-com crisis.

Figure 4 is the monthly chart of SP500 and NED from 1999 to 2004. It covers the period of the Dot-Com bubble burst and recovery. An all-time- high was presented in July 1999 with a NED of 0.27, a warning signal-five. The signal was followed by a 13% market slide in the next two months. Another signal-five appeared in December 1999 with an all-time-high accompanied with a NED value of 0.65, which led to a 10% market drop. The third signal-five presented in March 2000 was the strongest: the NED value was -0.35 at the market all-time-high when the dot-com bubble burst and the recession commenced (Odekon 2010).

One may ask why only the third signal-five led to the three-year recession. The reason is the follow-up intensified feedbacks among investors. In 2000, NEDs kept low after March, indicating a pessimistic view of price-takers. The morose sentiments influenced price-makers, and SP500 started to drop in October. The interaction and feedback between price-takers and price-makers led to a downtrend that can be identified and inferred by signal-two (November 2000, March 2001, September 2001, July 2002) and signal-four (December 2000, June, August 2001, March 2002) alternatively. The descending trend came to a stop between July and October 2002 when NED values locked at -1 with no new market lows. In the monthly chart, the reversal could be identified in June 2003 by signal-one. We can identify the trend reversal at a much earlier time using finer-time intervals. A signal-one appeared in the week of 4/21/2003 and on 3/17/2003, respectively.

### 6.2 The subprime crisis of 2008

**Figure 5:**
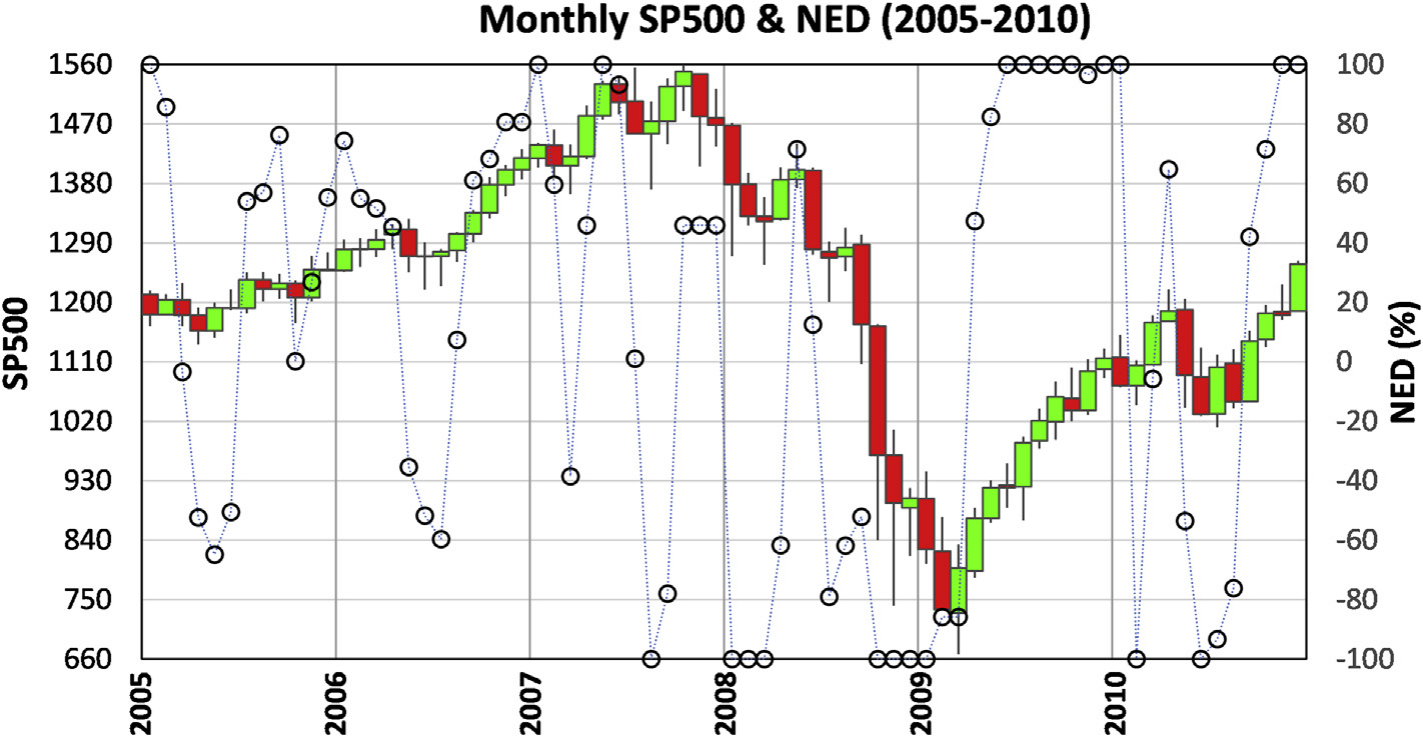
Monthly SP500 and NED for 2005–2010 covering the period of the onset and recovery of the 2008 subprime crisis.

Figure 5 is a monthly chart of SP500 and NED from 2005 to 2010. We can identify an uptrend from 2005 to August 2007 based on signal-one (October 2005, January 2006, January, March, and May 2007) and signal-three (July 2006, August 2007). A signal-five appeared in October 2007 when SP500 reached an all-time high with a NED value of 0.46, indicating price-takers' gloomy view about the market peak. Without the full support of price-takers for three months, SP500 started to fall, and a signal-two in January 2008 identified the trend reversal. A signal-four in April 2008 reveals that large sell-pressure in the order book by price-makers and the market doomed to go down. In July 2008, a signal-six hinted at a possible trend reversal. However, the turnaround did not go far, and the market kept moving down until January 2009. In March 2009, the SP500 was at the lowest level, but the NED increased from -1 to -0.83, flagging another signal-six. This time the reversal was successful, which was validated in February 2010 by a signal-three.

One may argue that many of the predictions can only be seen afterward. For example, given an upward reversal signal-six in July 2008, how do we know that the market would resume a downtrend in September? Furthermore, is it too late that confirming the reversal signal of March 2009 had to be 10 months later when the SP500 shot up 450 points (a 68% increase)? The answer lies in the shorter time scale chart.

**Figure 6:**
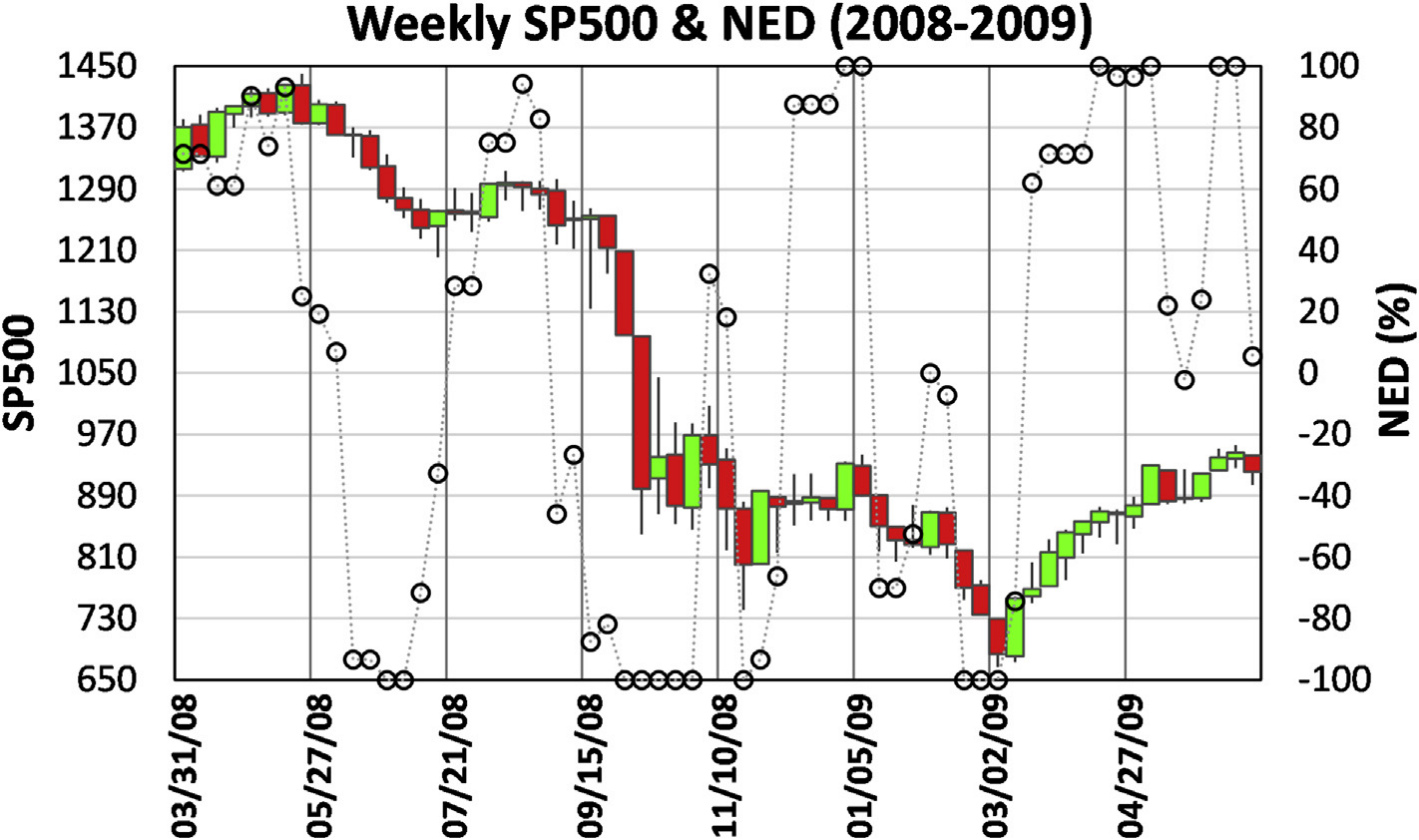
Weekly SP500 & NED for details of trend reversal process at the recovering stage of the subprime crisis.

Figure 6 is a weekly chart that helps to answer the questions. At the end of July 2008, when a signal-six presented in the monthly chart, the interaction detail between price-takers and price-makers is revealed in the weekly horizon. Initially, the SP500 and NED went up in the weeks of 7/28, 8/4, 8/11, conformed to the monthly signal. However, in the week started on August 18, the NED reached a ridge higher than the previous NED ridge, but the SP500 level was much lower than at that time. That is a signal-four warning of a downturn. A signal-two in the week of 9/15 confirmed the downtrend before the market significantly dropped. Now turn to the trend reversal in March 2009. A signal-one presented in the week of 5/4/2009, seven months before the monthly signal-three in February 2010. The daily signal (not shown here) would confirm the trend reversal by a signal-one as early as 3/11/2009 when SP500 just rose 8.2% from the lowest level of 666.79 on 3/6/2009.

## 7 Summary

The efficient market hypothesis that security prices fully reflect all available information is hard to contradict, given that the market is not easily beaten, although “the extreme version of the market efficiency hypothesis is surely false.” (Fama, 1991) This study shows that the time required for the price to fully reflect the information is dependent on the speed of information dissemination, which is recorded by NEDs. For short-lived information, the time required is in minutes, such as the Hillary Clinton email case on 10/28/2016 and Joe Biden's claim of tax raise on 4/24/2021 (not shown here). For long-lived information, the time needed is in days (Covid-19 virus spread 2/13–19, 2020) or even months (Tech bubble in 2000 and subprime crisis in 2008), consistent with the Kyle (1985) model.

Behavioral economists argue that markets are not efficient because market prices often depart far from fundamental values (Shiller, 1981; De Bondt and Thaler, 1990) due to irrational behaviors of investors under the influence of the market psychology (Kahneman and Tversky, 1979; Tversky and Kahneman, 1986; Simon, 1990; Rabin, 1998). This contradicts the statement that “in an efficient market at any point in time, the actual price will be a good estimate of its intrinsic value.” (Fama, 1965) Settling the debate between EMH and BFT is challenging because even definitions of market efficiency are different (Shefrin, 2008; Malkiel, 2000; Shleifer, 2000), and BFT does not have a behavioral price model^6^ because of the lack of measurements of investor behaviors.

The paper fills up the gap of measurements of investor behaviors without making assumptions about rational or irrational behaviors. Our model removes the implicit assumption of perfect competition in the model of Samuelson (1941, Eq. 11) so that market price changes are driven by behavior interactions of liquidity takers and liquidity providers (Bouchaud et al., 2004; Farmer, 2006). We show that news influences investor behaviors and their interactions to change market prices, which takes different times depending on the nature of the information. Other than political and economic news, price movements are information (Mantin and Gillen, 2011) that constantly impacts investor behaviors, which explains why business news alone can only explain a small fraction of market fluctuations (Cornell, 2013). The assumption underlying behavior analyses, and the six signals summarized in the paper is that the measured NED describes behaviors of liquidity-takers, which is validated by the market maker's inventory positions and the enhanced role of liquidity providers in the period of volatile markets.

We focus on academic issues of measurability of market excess demand and its explanatory power of the stock market daily fluctuations in the paper. The practitioners' interest in whether NED signals can help earn abnormal profits for different horizons is an ongoing investigation with promising preliminary results. For example, based on daily and weekly signals, one can obtain 46% and 75% returns by trading "long only" and "long and short", respectively, higher than the 16% return by the buy-and-hold strategy in the year 2020 as listed in Table 2 (buy and sell positions are closing SP500 levels of the day). Note that there are more signals than those listed in the table. For example, several signals 1 and 3 before signal 5 appeared on February 19 (Figure 3). Since those signals imply an uptrend movement, no action is needed to exit the "long" position. We will report our investigation in more detail in a follow-up paper.

**Table 2:** Profits earned based on B&H strategy and NED signals.

Date	Buy Position	Sell Position	Gain (Long)	Gain (Short)	NED Signal #
12/31/2019	3230.78				
2/19/2020		3286.15	55.37		5
3/24/2020	2447.33			838.82	6
9/16/2020		3385.49	938.16		4
10.28/2020	3271.03			114.46	3
12/31/2020		3756.07	485.04		
Profit using Buy and hold strategy = (3756.07–3230.78)/3230.78 = 0.1626					
Profit using NED signal (Long only) = (55.37 + 938.16+485.04)/3230.78 = 0.4577					
Profit using NED signal (Long and Short) = (55.37 + 938.16+485.04 + 838.82+114.46)/3230.78 = 0.7527					

Perfect competition, in which all agents are price-takers, is a fundamental and prevalent concept in macroeconomics despite criticism of it since the 19^th^ century, with its use defended by the claim that it is a good enough approximation for general phenomena, with the result that it is widely used in theoretical work (Stigler, 1957). We have shown that this empirical defense is inadequate when applied to the stock market, where it ignores the role of market makers and their interactions with price-takers.

Our study treats price-takers (Lee and Radhakrishna, 2000; Chakrabarty and Zhang, 2012) and price-makers (Cont et al., 2014; Gould and Bonart, 2016) as separate interacting agents. We fit the measured price-takers behaviors into an empirical function of price change so that real-time retrievals of NED at different horizons are possible. The summarized six signals of investor interactions and feedbacks between price-takers and price-makers are indicative of market structure and future directions of both short-term and long-term market movements. While the initial results are encouraging in predicting price reversals, a gap of the study is that NED cannot predict targets of price reversals except cases of market makers’ inventory positions at daily or intraday horizons. In addition, much work needs to be done (e.g., identifying rational or irrational investor behaviors, exploring more NED signals for foreseeing price movement directions, calculating statistics of fail and success rate of NED signals and profits earned at different horizons) for better understanding the cause and predictability of financial market fluctuations.

The datasets generated during the current study are available from the corresponding author on reasonable request.

## Declarations

### Author contribution statement

Qingyuan Han: Conceived and designed the experiments; Performed the experiments; Contributed analysis tools and data; Analyzed and interpreted the data; Wrote the paper.

Steve Keen: Analyzed and interpreted the data.

### Funding statement

This research did not receive any specific grant from funding agencies in the public, commercial, or not-for-profit sectors.

### Data availability statement

Data will be made available on reasonable request.

### Declaration of interests statement

The authors declare no conflict of interest.

### Additional information

No additional information is available for this paper.

## Footnotes

1 For EMH: Harry Markowitz (1990), Merton Miller (1990), William Sharpe (1990), Robert Merton (1997), Myron Scholes (1997), and Eugene Fama (2013), For BFT: Herbert Simon (1978), Daniel Kahneman (2002), Robert Shiller (2013), and Richard Thaler (2017).

2 http://hutong9.net/forum.php?mod=viewthread&amp;tid=430972, mouse right click for a poor google translation.

3 https://twitter.com/jasoninthehouse/status/792047597040971776.

4 https://www.propublica.org/article/burr-family-stock.

5 http://hutong9.net/forum.php?mod=viewthread&amp;tid=430972&amp;extra=&amp;page=19.

6 The father of the EMH, Eugene Fama once claimed, “*I'm probably the most important behavioral-finance person, because without me and the efficient-markets model, there is no behavioral finance. I still think there is no full-blown testable behavioral asset-pricing model*.” (Chicago Booth Review, 2016).
